# Effects of Empagliflozin and Lifestyle Intervention on Improving Body Weight and Other Metabolic Parameters in Atypical Antipsychotics-treated Patients with Schizophrenia Spectrum Disorders—A Double-blind Randomized Placebo-controlled Trial

**DOI:** 10.1093/schbul/sbag050

**Published:** 2026-05-09

**Authors:** Sin-Ying Wong, Sheung-Chun Chan, Yi-Man Flora Mo, Joshua Tsoh, Wing-Ho Oscar Wong, Tsun-Ming Yu, Sau-Man Sandra Chan, Hei-Ming Lai

**Affiliations:** Department of Psychiatry, Faculty of Medicine, The Chinese University of Hong Kong, c/o Ground Floor, Multicentre, Tai Po Hospital, 9 Chuen On Road, Tai Po, New Territories, Hong Kong SAR, China; Department of Psychiatry, Tai Po Hospital/Alice Ho Miu Ling Nethersole Hospital/North District Hospital, New Territories East Cluster, Hospital Authority, Hong Kong SAR, China; Department of Psychiatry, Faculty of Medicine, The Chinese University of Hong Kong, c/o Ground Floor, Multicentre, Tai Po Hospital, 9 Chuen On Road, Tai Po, New Territories, Hong Kong SAR, China; Department of Psychiatry, Tai Po Hospital/Alice Ho Miu Ling Nethersole Hospital/North District Hospital, New Territories East Cluster, Hospital Authority, Hong Kong SAR, China; Department of Psychiatry, Faculty of Medicine, The Chinese University of Hong Kong, c/o Ground Floor, Multicentre, Tai Po Hospital, 9 Chuen On Road, Tai Po, New Territories, Hong Kong SAR, China; Department of Psychiatry, Tai Po Hospital/Alice Ho Miu Ling Nethersole Hospital/North District Hospital, New Territories East Cluster, Hospital Authority, Hong Kong SAR, China; Department of Psychiatry, Faculty of Medicine, The Chinese University of Hong Kong, c/o Ground Floor, Multicentre, Tai Po Hospital, 9 Chuen On Road, Tai Po, New Territories, Hong Kong SAR, China; Department of Psychiatry, Shatin Hospital/Prince of Wales Hospital, New Territories East Cluster, Hospital Authority, Hong Kong SAR, China; Department of Psychiatry, Faculty of Medicine, The Chinese University of Hong Kong, c/o Ground Floor, Multicentre, Tai Po Hospital, 9 Chuen On Road, Tai Po, New Territories, Hong Kong SAR, China; Department of Psychiatry, Tai Po Hospital/Alice Ho Miu Ling Nethersole Hospital/North District Hospital, New Territories East Cluster, Hospital Authority, Hong Kong SAR, China; Department of Psychiatry, Faculty of Medicine, The Chinese University of Hong Kong, c/o Ground Floor, Multicentre, Tai Po Hospital, 9 Chuen On Road, Tai Po, New Territories, Hong Kong SAR, China; Department of Psychiatry, Faculty of Medicine, The Chinese University of Hong Kong, c/o Ground Floor, Multicentre, Tai Po Hospital, 9 Chuen On Road, Tai Po, New Territories, Hong Kong SAR, China; Department of Psychiatry, Tai Po Hospital/Alice Ho Miu Ling Nethersole Hospital/North District Hospital, New Territories East Cluster, Hospital Authority, Hong Kong SAR, China; Department of Psychiatry, Faculty of Medicine, The Chinese University of Hong Kong, c/o Ground Floor, Multicentre, Tai Po Hospital, 9 Chuen On Road, Tai Po, New Territories, Hong Kong SAR, China; Department of Chemical Pathology, Faculty of Medicine, The Chinese University of Hong Kong, Sha Tin, Hong Kong SAR, China; Li Ka Shing Institute of Health Sciences, The Chinese University of Hong Kong, Sha Tin, Hong Kong SAR, China

**Keywords:** sodium-glucose co-transporter 2 inhibitors, empagliflozin, schizophrenia spectrum disorders, atypical antipsychotics, body weight, metabolic syndrome

## Abstract

**Background and Hypothesis:**

Patients with schizophrenia spectrum disorders have elevated cardiovascular risk with obesity and deranged metabolic profiles associated with antipsychotic usage, bearing significant tolls on chronic morbidity and mortality. The present study aims to evaluate the efficacy and tolerability of sodium-glucose co-transporter 2 inhibitors (SGLT2i) in improving weight control and metabolic profile in atypical antipsychotics-treated patients with schizophrenia spectrum disorders.

**Study Design:**

This double-blind, randomized, placebo-controlled trial studied overweight or obese adults with schizophrenia spectrum disorders (non-diabetic or pre-diabetic) who were taking atypical antipsychotics. Participants recruited from a specialist clinic of a regional public service were randomized to receive empagliflozin or placebo for 16 weeks, in combination with standardized lifestyle intervention through psychoeducation. The primary outcomes were changes in body weight and body mass index (BMI). Changes in other metabolic parameters were examined as secondary outcomes. Linear mixed models were used to study the differences in outcomes between the active and placebo arms.

**Results:**

Of the 52 patients undergoing randomization, the SGLT2i group had significant reductions in body weight (−1.53 kg; 95% CI, −2.60 to −0.47), BMI (−0.53 kg/m^2^; 95% CI, −0.91 to −0.15), fasting glucose (−0.40 mmol/L; 95% CI, −0.65 to −0.15), and glycated hemoglobin A1c (−0.11%; 95% CI, −0.21 to −0.02) compared with the placebo group (*P*-values <.05). Adverse events and dropout rates were similar between the 2 groups.

**Conclusions:**

The combined approach of empagliflozin and lifestyle intervention improved weight control and glucose metabolism among patients with schizophrenia spectrum disorders receiving atypical antipsychotics.

## Introduction

People with schizophrenia spectrum disorders have a curtailed life expectancy of at least 14-16 years,^1^ with cardiovascular disease being one of the major potentially avoidable causes.^2^ Obesity and other metabolic abnormalities, prevalent in people with schizophrenia spectrum disorders, are important risk factors. Atypical antipsychotics are increasingly used and effective for treating schizophrenia spectrum disorders.^3^^,^^4^ Among the atypical antipsychotics, clozapine, olanzapine, quetiapine, risperidone, and paliperidone are particularly known for their high propensity for weight gain and metabolic derangements.^5–7^ The consequential effects of weight gain and medical comorbidities include medication non-adherence, reduced self-esteem, demoralization, and increased social stigma—issues that are already pronounced in schizophrenia spectrum disorders.^8^

The mechanism of antipsychotic-associated weight gain has not been fully understood, although studies have proposed the roles of central histaminergic H1 and serotoninergic 5-HT2C receptors antagonisms.^7^^,^^9^ Chronic inflammatory and immunological abnormalities are also postulated to be contributing factors,^10^^,^^11^ alongside a complex interplay of other genetic, biological, environmental, and lifestyle factors.

Lifestyle interventions have shown efficacy in helping schizophrenic patients to control their weights and are the first-line management for weight gain in patients with schizophrenia spectrum disorders on antipsychotics.^12–14^ However, barriers to effective implementation of lifestyle intervention alone might include impaired cognitive functions, motivational deficits, economic and geographical disadvantage in food, and facilities access among schizophrenic patients.^15^

Recent studies shed light on some of the adjunctive anti-diabetic drugs in improving weight control and metabolic parameters, especially insulin resistance,^14^^,^^16–18^ Metformin is by far the most-studied agent for reducing weight in patients on atypical antipsychotics. It shows positive results as early as 12 weeks, and it is most effective when combined with lifestyle interventions.^14^^,^^16^ Yet, its effects often plateau by 12 weeks with only partial reversal of the weight accrued during antipsychotic treatment.^19^ Its twice-daily dosing and associated gastrointestinal side effects also make it a less attractive choice. Recent studies showed a prominent effect of glucagon-like peptide-1 (GLP-1) receptor agonists, delivered as weekly injections, in weight control among patients with schizophrenia spectrum disorders who are on atypical antipsychotics.^18^^,^^20^^,^^21^^,^^22^ Notwithstanding the limited access of this class of highly priced novel agents to service users with schizophrenia spectrum disorders, there have been rising concerns about adverse events of GLP-1, such as gastrointestinal side effects (especially nausea, which occurs up to 60%),^20^ association with pancreatitis,^23^ thyroid cancer,^24^ and suicidality^25^ against the explosive popularity in the anti-obesity market.^26^

Sodium-glucose co-transporter 2 inhibitors (SGLT2i) are a novel class of oral anti-diabetic medications approved for treating type 2 diabetes. They specifically target and block the sodium-glucose co-transporter 2 in the renal proximal tubule, thereby increasing urinary glucose excretion and subsequent caloric and weight loss. Their weight-losing effect expands to non-diabetic individuals with well-demonstrated tolerability and safety.^27^^,^^28^ They are also shown to improve blood pressure control,^29^ and are convenient to use due to their oral form and once-daily dosing. The convenient dosing schedule, safety, and effectiveness of SGLT2i lend themselves to being a candidate agent in the clinical care of schizophrenia. However, there are limited data from clinical and preclinical studies on the effects of SGLT2i on antipsychotic-related weight gain in schizophrenia spectrum disorders, both globally and among Asian countries.^30^ A recent randomized controlled trial conducted in Iran demonstrated the positive effect of empagliflozin, one of the SGLT2i, in improving body weight, body mass index (BMI), waist circumference, and systolic blood pressure (SBP) in patients taking atypical antipsychotics.^31^ However, the study population was heterogenous, including all psychiatric disorders and patients with and without diabetes mellitus on various combinations of diabetic medications. Nonetheless, the putative mechanisms underpinning weight reduction by SGLT2i are complex, encompassing urinary glucose loss and thus negative caloric balance, improvement in mitochondrial function, polarization of macrophages from M1 to M2, and lipolysis.^32^ A recent preclinical animal study has shown a gender-specific weight attenuation effect of emagliflozin in female Wister rats treated with olanzapine.^33^

This study reports the outcomes of a 16-week randomized, double-blind, placebo-controlled trial that tested the efficacy of lifestyle intervention augmented with 10 mg of empagliflozin daily in reducing weight and improving metabolic parameters among obese or overweight non-diabetic patients diagnosed with schizophrenia spectrum disorders and receiving atypical antipsychotics.

## Methods

### Participants

Eligible participants were adults aged 18-64 years diagnosed with schizophrenia and other psychotic disorders according to the *Chinese-bilingual Structured Clinical Interview for Diagnostic and Statistical Manual of Mental Disorders, Fourth Edition Axis I (SCID-I), patient version*^34^ except for psychotic disorder due to a general medical condition and substance-induced psychotic disorder. Other enrollment criteria included that participants were Asians with a BMI of 23 or greater^35^; had been on at least one of the targeted antipsychotic agents—clozapine, olanzapine, quetiapine, risperidone, or paliperidone—for a minimum of 6 months; and were clinically stable (the Clinical Global Impression Scale—Severity [CGI-S] ≤ 4).^36^ The specified BMI for the inclusion criteria, notably lower than the universal cut-off (BMI > 25), was based on the revised definition recommended by World Health Organization for the Asia-Pacific region, ie, the cut-offs for overweight is ≥23.0 kg/m^2^ and obesity ≥25.0 kg/m^2^  ^35^ according to the risk prediction studies on metabolic syndrome and cardiovascular diseases in this region.^37^ Main exclusion criteria included having a psychiatric diagnosis other than schizophrenia spectrum disorder, active substance abuse, diabetes mellitus, serious physical illness with significant impact on weight control, or recurrent urinary tract infection. A detailed list of all inclusion and exclusion criteria is presented in Table S1.

Participants were recruited from psychiatric outpatient clinics and psychiatric day hospitals in the New Territories East Cluster (NTEC) in Hong Kong between November 2023 and April 2024. The trial was approved by the Joint Chinese University of Hong Kong—NTEC Clinical Research Ethics Committee and the Drug Office of the Department of Health (Ref. No.: 2023.256). All participants provided written informed consent, and the study was conducted in accordance with the Declaration of Helsinki and Good Clinical Practice.

### Study Design

Eligible participants were randomly assigned to 16 weeks of treatment with oral empagliflozin or placebo. A standardized lifestyle intervention was administered individually to all participants from either treatment group. Participants were randomized in blocks of 6 and stratified by sex into 2 groups, male and female, respectively, to ensure even distribution of participants. To ensure concealment of the treatment assignment, randomization was conducted independently by a psychiatrist (C.S.M.S.) who was not involved in the enrollment and assessment.

### Pharmacological Intervention

Empagliflozin (Jardiance) was administered in a double-blind, placebo-controlled fashion. Participants took 10 mg of empagliflozin or a placebo daily. They were given record sheets to log their daily intake of trial medication and pillboxes to encourage their adherence.

Participants continued their regular psychiatric care without interference, maintaining their specified atypical antipsychotics with a maximum allowable dosage adjustment of 25%. Otherwise, they would be withdrawn. Any dosage adjustments were carefully documented. Other medications, such as antidepressants, hypnotics, and mood stabilizers, were allowed if clinically indicated.

Pill counts were used to assess adherence during assessments. Satisfactory adherence was defined as taking more than 80% of the study drug dosage during the trial. Participants who were not adhering to the study medication were advised on the importance of taking the prescribed dosage.

### Lifestyle Intervention

The lifestyle intervention was in the form of psychoeducation administered by the lead author (W.M.S.Y.), who was blinded to the group assignment of the study participants, focusing on the roles of diet and physical activity in managing weight. During each assessment, participants received one-on-one individualized sessions, covering topics such as healthy eating, exercise, and calorie counting, with supplemental resources.^38–40^ All participants charted a 3-day food diary before each assessment. At the assessments, their food diaries were reviewed, any nutritional questions were clarified, their physical activity levels were assessed, and their difficulties in weight management were addressed.

### Measures

Baseline assessments included clinical and demographic characteristics, physical examination including anthropometric measurements (weight, height, waist circumference, SBP, and diastolic blood pressure [DBP]), and laboratory examinations. At baseline and at each assessment, CGI-S and Positive and Negative Syndrome Scale (PANSS)^41^ were used for evaluating and monitoring psychiatric symptoms; the average daily caloric intake of participants was calculated from their 3-day diet records; and their physical activity levels were evaluated using the International Physical Activity Questionnaire—Short Form.^4^ The laboratory examinations at baseline included blood counts, liver and renal function, thyroid function, fasting glucose, glycated hemoglobin A1c (HbA1c), low-density lipoprotein (LDL), high-density lipoprotein (HDL), total triglycerides (TG), electrocardiogram, urine pregnancy test, and urinary culture. All clinical assessments were administered by the lead author (W.M.S.Y.).

The anthropometric measurements were reevaluated at week 8 and week 16, while the laboratory examinations were reevaluated at week 16.

The primary outcomes were changes in body weight and BMI from baseline to week 16. Secondary outcomes included changes in waist circumference, SBP, DBP, fasting glucose, HbA1c, LDL, HDL, and TG. The tolerability and safety of empagliflozin were evaluated with regard to the incidence of side effects and adverse events.

### Sample Size Estimation and Statistical Analysis

A sample size of 52 participants (26 in each group) was estimated a priori based on the difference in mean body weight change in a recently published study by Santos-Gallego et al.,^28^ which has a population characteristic most closely resembling our target population and has a similar study design; and the SD for weight change in lifestyle + placebo group by Wu et al.^14^ The sample size was estimated at a power of 80%, a significance level (*α*) of 0.05 and a 5%-10% drop-out rate observed in similar studies.^14^^,^^19^ Statistical analyses were performed using the Statistical Package for Social Science (SPSS) version 27 (SPSS Inc., Chicago, Illinois). Data analysis was conducted according to the intention-to-treat (ITT) principle, in which all randomized participants who received at least one dose of the study medication were included. Missing data was addressed using linear mixed models. For comparison of baseline demographics and clinical characteristics between the 2 groups, *t*-tests or Mann–Whitney *U* tests were used for comparing continuous data, while *X^2^* analysis, and Fisher’s exact tests were used for comparing proportions whenever appropriate. Linear mixed models with corresponding baseline values as covariates were used to analyze changes in outcomes over time between both groups. Fixed effects included group (SGLT2i group vs placebo group), time (baseline, week 8, week 16), and their interaction (group × time), while random intercepts were included to account for individual variability within the study participants. The group × time interaction effect was considered to evaluate the efficacy of the combined SGLT2i-lifestyle intervention approach on the outcome measures. The statistical significance was referred to a 2-tailed *P* < .05 for all tests unless otherwise specified. Post hoc sensitivity analyses and exploratory analyses (Tables in Supplement Materials) were conducted to evaluate the robustness of the primary analysis and identify subgroup effects, respectively.

## Results

### Study Population and Baseline Characteristics

**Figure 1:**
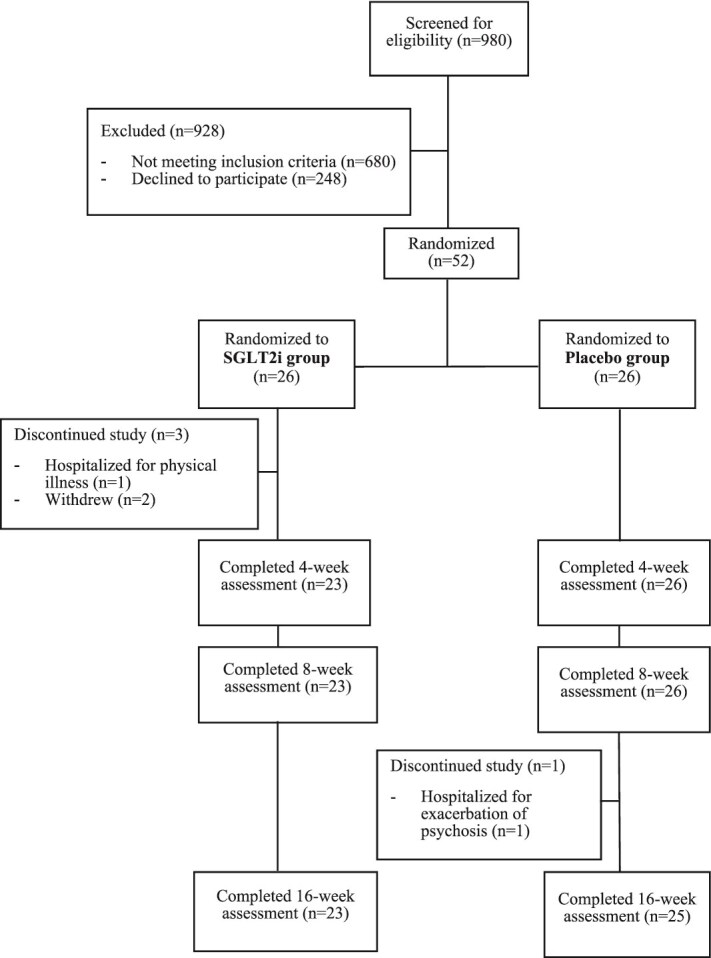
CONSORT diagram.

Of 52 eligible and consenting participants, 26 participants were randomly assigned to each of the treatment groups (Figure 1). A total of 48 participants (92.31%) completed the 16-week trial: 23 (88.46%) in the SGLT2i group and 25 (96.15%) in the placebo group. The demographic or clinical characteristics and baseline measurements did not differ significantly between groups (Table 1).

**Table 1:** Baseline Demographics and Clinical Characteristics

	SGLT2i group	Placebo group	Total	Statistics	*P*
	(*n* = 26)	(*n* = 26)	(*n* = 52)		
Age, mean (SD), year	42.88 (9.86)	41.50 (11.48)	42.19 (10.62)	0.47^a^	.64
Gender, *n* (%)				0.00^b^	1.00
Male	13 (50)	13 (50)	26 (50)		
Female	13 (50)	13 (50)	26 (50)		
Education, *n* (%)				1.94^c^	.64
Tertiary	3 (11.5)	3 (11.5)	6 (11.5)		
Upper secondary/diploma	16 (61.5)	18 (69.2)	34 (65.4)		
Lower secondary	7 (26.9)	4 (15.4)	11 (21.2)		
Primary	0 (0)	1 (3.8)	1 (1.9)		
Diagnosis, *n* (%)				5.51^c^	.051
Schizophrenia	25 (96.2)	21 (80.8)	46 (88.5)		
Schizoaffective disorder	0 (0)	3 (11.5)	3 (5.8)		
Acute and transient psychosis	0 (0)	2 (7.7)	2 (3.8)		
Delusional disorder	1 (3.8)	0 (0)	1 (1.9)		
Duration of diagnosis, mean (SD), year	12.11 (8.41)	14.23 (9.62)	13.17 (9.01)	−0.85^a^	.40
Targeted antipsychotic agent(s), *n* (%)				7.93^c^	.58
Clozapine	8 (30.8)	9 (34.6)	17 (32.7)		
Olanzapine	6 (23.1)	7 (26.9)	13 (25.0)		
Risperidone	3 (11.5)	1 (3.8)	4 (7.7)		
Paliperidone	5 (19.2)	3 (11.5)	8 (15.4)		
Quetiapine	1 (3.8)	3 (11.5)	4 (7.7)		
Clozapine + paliperidone	1 (3.8)	0 (0)	1 (1.9)		
Clozapine + quetiapine	0 (0)	1 (3.8)	1 (1.9)		
Olanzapine + paliperidone	0 (0)	2 (7.7)	2 (3.8)		
Risperidone + quetiapine	1 (3.8)	0 (0)	1 (1.9)		
Paliperidone + quetiapine	1 (3.8)	0 (0)	1 (1.9)		
Dose of targeted antipsychotics, median (IQR), DDD^f^	1.17 (1.28)	1.21 (0.70)	1.21 (1.25)	369.00^e^	.57
Family history of diabetes, *n* (%)	10 (38.5)	10 (38.5)	20 (38.5)	0.00^b^	1.00
Family history of obesity, *n* (%)	11 (42.3)	11 (42.3)	22 (42.3)	0.00^b^	1.00
Active smoker, *n* (%)	8 (30.8)	6 (23.1)	14 (26.9)	0.39^b^	.53
Active drinker, *n* (%)	3 (11.5)	0 (0)	3 (5.8)	4.34^d^	.24
Body weight, median (IQR), kg	76.85 (8.49)	75.38 (19.4)	76.70 (13.74)	320.00^e^	.74
BMI, median (IQR), kg/m^2^	28.54 (5.44)	28.87 (6.95)	28.85 (5.60)	365.50^e^	.62
Waist circumference, mean (SD), cm	101.43 (8.91)	103.83 (10.52)	102.63 (9.73)	−0.89^a^	.38
SBP, mean (SD), mm Hg	127.46 (16.48)	126.54 (14.49)	127.00 (15.37)	0.22^a^	.83
DBP, median (IQR), mm Hg	76.00 (16.75)	81.00 (8.50)	79.50 (10.75)	377.50^e^	.47
Glucose metabolism					
Fasting plasma glucose level, mean (SD), mmol/L	5.63 (0.52)	5.58 (0.45)	5.60 (0.48)	0.37^a^	.71
HbA1c, mean (SD), %	5.61 (0.38)	5.69 (0.37)	5.65 (0.37)	−0.82^a^	.42
Cholesterol level					
Total, mean (SD), mmol/L	5.30 (0.81)	5.00 (0.69)	5.14 (0.76)	1.49^a^	.14
LDL, mean (SD), mmol/L	3.29 (0.63)	2.98 (0.68)	3.14 (0.66)	1.68^a^	.10
HDL, mean (SD), mmol/L	1.32 (0.25)	1.23 (0.23)	1.27 (0.24)	1.38^a^	.17
TG, median (IQR), mmol/L	1.60 (0.92)	1.55 (0.93)	1.55 (0.88)	347.00^e^	.87
Psychiatric ratine scales CGI-S, median (IQR), score	2.00 (1.00)	2.00 (2.00)	2.00 (1.00)	338.00^e^	1.00
PANSS (total), mean (SD), score	49.50 (7.85)	47.69 (8.71)	48.60 (8.26)	0.79^a^	.44
PANSS (negative scale), mean (SD), score	14.15 (3.64)	13.27 (4.53)	13.71 (4.09)	0.78^a^	.44
Lifestyle measures Average daily caloric intake, mean (SD), kcal	1886.15 (500.72)	1921.47 (431.86)	1903.81 (463.30)	−0.27^a^	.79
Physical activity level, median (IQR), MET-min/week	693.00 (1103.25)	471.00 (1138.50)	658.00 (1089.00)	333.00^e^	.93

^a^Student’s *t-test*,

^b^ *ꭓ* ^2^ test,

^c^Fisher–Freeman–Halton exact test,

^d^Fisher’s exact test,

^e^Mann–Whitney *U* test,

^f^Daily defined dose (DDD).

Abbreviations: BMI, body mass index; DBP, diastolic blood pressure; CGI-S, clinical global impression scale—severity; HbA1c, glycated hemoglobin A1c; HDL, high-density lipoprotein; IQR, inter-quartile range; MET, metabolic equivalents of task; LDL, low-density lipoprotein; SBP, systolic blood pressure; SD, standard deviation; TG, total triglycerides.

Among the 48 participants who finished the entire study, 93.75% adhered to the study medication, defined as taking more than 80% of the study drug dosage prescribed for the interval. All nonadherent participants belonged to the SGLT2i group. Data obtained in outcome measures for all participants were included in the final analysis and were addressed according to the ITT principle.

### Primary Outcomes: Changes in Body Weight and BMI

The linear mixed model showed that significant overall effects of group-by-time interaction (*F* [1, 46.46] = 5.14, *P* = .028), group (*F* [1, 46.99] = 4.89, *P* = .032), and time (*F* [1, 46.46] = 16.75, *P* = <.001) were found for body weight. The model on BMI showed consistent results with a significant overall effect of significant group-by-time interaction (*F* [1, 46.43] = 4.91, *P* = .032), group (*F* [1, 47.01] = 4.50, *P* = .039), and time (*F* [1, 46.43] = 15.59, *P* = <.001).

**Figure 2:**
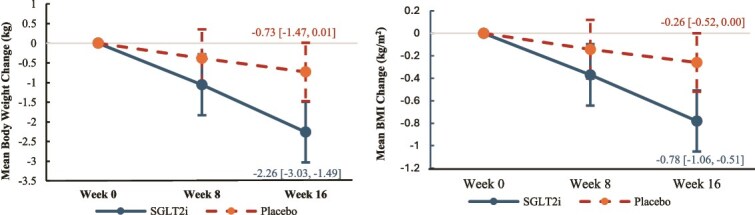
Changes of body weight and BMI of SGLT2i group vs placebo group. Error bars represent 95% confidence interval.

Figure 2 and Table 2 showed that the SGLT2i group had a significantly greater reduction in body weight compared to the placebo group at week 16 ($\hat{\beta}$ = −1.53 kg [−2.60, −0.47], *t* (60.07) = −2.87, *P* = .006; $\hat{\beta}$ = estimated fixed effects coefficient) but not at week 8.

**Table 2:** Changes in End Points from Baseline to Week 16

Variables	SGLT2i group (*n* = 26)	Placebo group (*n* = 26)	SGLT2i group–placebo group Estimated mean difference [95% CI]	*P*-value
Body weight, kg	−2.26 [−3.03, −1.49]	−0.73 [−1.47, 0.01]	−1.53 [−2.60, −0.47]	**.006** ^*^
BMI, kg/m^2^	−0.78 [−1.06, −0.51]	−0.26 [−0.52, 0.00]	−0.53 [−0.91, −0.15]	**.007** ^*^
Waist circumference, cm	−2.60 [−4.37, −0.82]	−2.32 [−4.04, −0.60]	−0.28 [−2.76, 2.21]	.823
SBP, mm Hg	−3.54 [−9.05, 1.97]	−5.45 [−10.82, −0.08]	1.91 [−5.78, 9.60]	.622
DBP, mm Hg	−0.96 [−4.37, 2.45]	−4.12 [−7.44, −0.80]	3.16 [−1.60, 7.93]	.190
Fasting plasma glucose, mmol/L	−0.42 [−0.60, −0.24]	−0.02 [−0.20, 0.15]	−0.40 [−0.65, −0.15]	**.002** ^*^
HbA1c, %	−0.03 [−0.10, 0.04]	0.08 [0.02, 0.15]	−0.11 [−0.21, −0.02]	**.019** ^*^
LDL, mmol/L	−0.15 [−0.36, 0.06]	−0.22 [−0.43, −0.02]	0.07 [−0.22, 0.37]	.629
HDL, mmol/L	0.04 [−0.03, 0.11]	−0.03 [−0.10, 0.04]	0.07 [−0.03, 0.17]	.166
TG, mmol/L	−0.07 [−0.35, 0.21]	0.03 [−0.24, 0.31]	−0.10 [−0.49, 0.29]	.609

^*^ *P* < .05

Abbreviations: BMI, body mass index; DBP, diastolic blood pressure; CGI-S, clinical global impression scale—severity; HbA1c, glycated hemoglobin A1c; HDL, high-density lipoprotein; LDL, low-density lipoprotein; SBP, systolic blood pressure; TG, total triglycerides.

Figure 2 and Table 2 showed that the SGLT2i group had correspondingly significantly greater reduction in BMI compared to the placebo group at week 16 ($\hat{\beta}$ = −0.53 kg/m^2^ [−0.91, −0.15], *t* [60.16] = −2.77, *P* = .007) but not at week 8.

### Secondary Outcomes: Changes in Waist Circumference, SBP and DBP, Fasting Glucose, HbA1c, and Lipid Levels

Overall, none of the linear-mixed models showed significant group-by-time interactions for all secondary outcome measures. There was a significant overall time effect (*F* [1, 46.38] = 5.11, *P* = .028) for waist circumference, but there was no significant group-by-time interaction or group effect detected. There was also no significant group-by-time interaction, time, or group effect detected for systolic and DBP.

**Figure 3:**
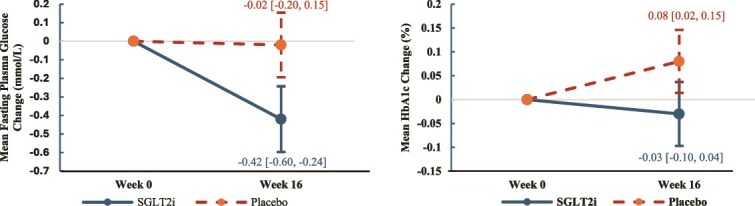
Changes in glucose metabolic parameters of SGLT2i group vs placebo group. Error bars represent 95% confidence interval.

While there were no significant effects for LDL, HDL, and TG, a significant group effect was observed in the reduction of fasting plasma glucose between the groups (*F* [1, 46] = 10.25, *P* = .002). Table 2 and Figure 3 showed the post-intervention fasting plasma glucose level was significantly reduced in the SGLT2i group compared to the placebo group ($\hat{\beta}$ = −0.40 mmol/L [−0.65, −0.15], *t* [46] = −3.20, *P* = .002). Similar results were observed in HbA1c, for which a significant group effect was observed (*F* [1, 46] = 5.96, *P* = .019). Post-intervention HbA1c level was significantly reduced in the SGLT2i group compared with the placebo group ($\hat{\beta}$ = −0.11% [−0.21, −0.02], *t* [46] = −2.44, *P* = .019) (Figure 3 and Table 2).

Lastly, it was notable that there was a significant time effect on subjects’ physical activity levels (Tables S2 and S3). Otherwise, no other effects or differences were detected in physical activity levels or caloric intake. The results remained largely similar in all primary and secondary outcomes in post hoc sensitivity analyses excluding outliers or including age, change in caloric intake, and physical activity levels as additional covariates (Tables S4 and S5).

### Subgroup Analyses

In exploratory subgroup analyses (Tables S6-S8), the reduction from baseline to week 16 outcomes in terms of fasting glucose and HbA1c among 3 subgroups was significant, ie, pre-diabetic (*P* = .025; *P* = .032), obese (*P* = .007; *P* = .033), and olanzapine/clozapine groups (*P* = .004; *P* = .020). In terms of body weight and BMI changes from baseline to week 16, the pre-diabetic group (*P* = .003; *P* = .004) and obese group (*P* = .009, *P* = .016) also showed statistically significant reductions.

### Adverse Events

There were no significant differences in the frequency and types of adverse events reported between the 2 groups (Table 3). The majority of the reported adverse events were mild. All the urinary symptoms reported were verified with negative urinary culture results. There was no genitourinary tract infection, hypoglycemia, or ketoacidosis detected during the trial. Two serious adverse events resulted in withdrawals from the trial. One participant from the SGLT2i group was hospitalized due to infectious mononucleosis necessitating hospitalization, whereas another in the placebo group experienced exacerbation of psychosis, leading to repeated hospital admissions. The drop-out rates did not exhibit a significant difference between the 2 groups.

**Table 3:** Adverse Events and Serious Adverse Events

	SGLT2i group	Placebo group	
	(*n* = 26) (%)	(*n* = 26) (%)	*P*
Dropouts	3 (11.5)	1 (3.8)	.61^b^
Any adverse events or serious adverse events	8 (30.8)	5 (19.2)	.34^a^
Gastrointestinal system			
Nausea	1 (3.8)	0 (0)	1.00^b^
Diarrhea	1 (3.8)	0 (0)	1.00^b^
Constipation	0 (0)	2 (7.7)	.49^b^
Cardiovascular system			
Palpitation	1 (3.8)	0 (0)	1.00^b^
Urinary system			
Dysuria	1 (3.8)	1 (3.8)	1.00^b^
Polyuria	1 (3.8)	0 (0)	1.00^b^
Nervous system			
Fatigue	2 (7.7)	0 (0)	.49^b^
Dizziness	1 (3.8)	1 (3.8)	1.00^b^
Respiratory system			
Dyspnea	1 (3.8)	1 (3.8)	1.00^b^
Others			
Hair loss	1 (3.8)	0 (0)	1.00^b^
Serious adverse events			
Total no.	1 (3.8)	1 (3.8)	1.00^b^
Somatic disease			
Infectious mononucleosis	1 (3.8)	0 (0)	1.00^b^
Psychiatric disease			
Admission to hospital for worsening of psychosis	0 (0)	1 (3.8)	1.00^b^

^a^ *ꭓ* ^2^ test,

^b^Fisher’s exact test.

## Discussion

This is a double-blind, randomized, placebo-controlled study that evaluated the efficacy and safety of combining empagliflozin with lifestyle intervention on improving weight control in obese or overweight patients with non-diabetic/pre-diabetic schizophrenia spectrum disorder treated with atypical antipsychotics. In this 16-week study, we found statistically significant differences in the reduction of mean body weight, BMI, fasting plasma glucose, and HbA1c in the SGLT2i group compared to the placebo group, but there were no differences observed in weight circumference, SBP and DBP, LDL, HDL, and TG levels. These results suggest that combined empagliflozin-lifestyle intervention can reduce weight and improve glucose metabolism in this specific population. Our study verified that empagliflozin treatment is safe and well tolerated, as evidenced by comparable drop-out rates and adverse events between the groups. There was no causal relationship between the study drug and the severe adverse events.

The significant body weight difference between the groups at week 16, as opposed to week 8, implies a cumulative and progressive effect of the treatment, suggesting the potential for further weight reduction with a longer treatment period. At week 16, when the most pronounced weight difference was observed, the SGLT2i group showed a significantly greater reduction in body weight compared to the placebo group, with an estimated mean difference of −1.53 kg (95% CI [−2.60, −0.47], *P* = .006). This result is consistent with existing evidence regarding the effect of SGLT2i on weight reduction in non-diabetic populations^28^^,^^29^ and patients taking antipsychotics.^31^ Additionally, the significant difference in the reduction of fasting blood glucose levels suggests that the empagliflozin-lifestyle intervention effectively halts the progressive deterioration of glucose metabolism in the study population.

Nevertheless, the combined empagliflozin and lifestyle intervention did not lead to significant improvements in metabolic parameters beyond changes in body weight, BMI, and glucose metabolism. While some previous studies have demonstrated modest reductions in systemic blood pressure with SGLT2 inhibitors,^29^^,^^31^^,^^42^ this was not the primary focus of the current analysis. The lack of observed significant changes in other metabolic outcomes may be partly due to the limited sample size and short duration of follow-up. Therefore, larger and longer-term studies are warranted to further investigate the multidimensional effects of empagliflozin and lifestyle interventions on metabolic health, including blood pressure and other cardiometabolic parameters.

The post hoc exploratory analyses should be interpreted with caution. The statistically significant reductions in body weight and glucose metabolism from baseline to week 16 among the sub-groups (with obesity, pre-diabetic state, medicated with clozapine/olanzapine) were observed in under-powered sub-samples undergoing multiple comparisons. The observed results are thus inconclusive. Future studies should include adequately powered clinical subgroups to provide more clinical evidence on patient selection for empagliflozin-lifestyle intervention.

Taken together, this study’s main findings should be interpreted considering the following methodological limitations. First and foremost, the generalizability of the study’s findings to other non-Asian populations should be cautioned, given the inherent differences in the clinical definition of obesity and overweight as well as lifestyle and dietary habits differing across cultures and ethnicities. It also follows that clinical subjects were suffering from schizophrenia spectrum disorders that encompass a wide range of mental illnesses treated with various psychotropic regimes, resulting in inevitable clinical heterogeneity. In terms of the clinical utility, it is premature to recommend the treatment in clinical practice, as we cannot determine if the improved body weight and glucose metabolism can be maintained or further improved beyond the 16-week trial period, considering the cumulative and progressive effects observed. A knowledge gap exists on whether the short-term (16-week) clinical benefits in body weight and glucose metabolism could be sustained after patients stop taking empagliflozin or engaging in the lifestyle interventions. Further, participants’ pre-medicated baseline body weight and the subsequent weight gain after using atypical antipsychotics were not retrievable from the generic clinical record, precluding subsequent inclusion of this potential confounder for statistical adjustment in the mixed-linear models. Possible measurement biases may be attributable to recall bias in the self-report on diet and physical activity levels and the inaccuracy in measuring medication adherence solely through pill counts.

Notwithstanding the inherent methodological limitations, the study’s robustness was supported by having a high protocol adherence to random participant assignment, double blinding, inclusion of covariates in the linear-mixed model, block randomization design, low attrition, post hoc sensitivity analyses. The low attrition in this special patient group with schizophrenia spectrum disorder is unexpected, and is plausibly related to several inherent characteristics of the treatment setting that favored the retention in the study protocol. First, the specialist clinic was accessible to a geographically small local neighborhood where the patients resided in. Aside, patients were recruited by their treating psychiatrists who identified that they had general health concerns and were motivated to participate. Given the low attrition and the comparable drop-out rates from both arms, no sensitivity analysis was therefore in this regard.

Taken together, findings of this study not only provide preliminary evidence on the short-term efficacy and safety of the intervention, but also highlight the need for further research into the use of SGLT2i either alone or in combination with lifestyle intervention for managing weight in the psychiatric population. The observed cumulative and progressive effects in this study underscore the necessity for additional research to ascertain the sustainability of the effects on weight control and glucose metabolism. This includes investigating the long-term impact of continued use as well as the sustained effects following the cessation of treatment. Future studies exploring the clinical efficacy of different types of SGLT2i, dosages, phases of initiation, the population who derives the best outcome, and the effects on other psychiatric diseases (such as bipolar affective disorder), could enhance precision in the proposed treatment algorithm. Furthermore, conducting comparative studies, such as head-to-head examinations with the best-studied comparators like metformin or other forms of lifestyle interventions, would also be beneficial.^16^^,^^17^ Other potential research areas include its effects on cognitive and psychiatric symptoms, as well as the underlying mechanisms for their weight-loss effect with respect to the purposed mechanisms of weight gain in atypical antipsychotic users. In particular, SGLT2i are well-proven to have anti-inflammatory properties and reduce oxidative stress.^43^ Its relationship with chronic inflammation in schizophrenia and obesity is also worth further understanding.^10^^,^^11^ Lastly, while this study does not definitively establish whether the proven cardiovascular benefits of SGLT2i can be extended to patients with schizophrenia spectrum disorder, the preliminary results are promising and give a heuristic value for future exploration in this area.

## Conclusion

In overweight or obese non-diabetic/pre-diabetic Asian patients with schizophrenia spectrum disorder, the combination of empagliflozin 10 mg daily and lifestyle intervention as adjunctive treatment to atypical antipsychotics was a safe, well-tolerated, and effective pharmacologic intervention. Empagliflozin-lifestyle intervention was superior in inducing weight loss and improving glucose metabolism than lifestyle intervention alone.

## Supplementary Material

sbag050_Supplementary_material
