# Supplementary material for: Effects of Empagliflozin and Lifestyle Intervention on Improving Body Weight and Other Metabolic Parameters in Atypical Antipsychotics-treated Patients with Schizophrenia Spectrum Disorders—A Double-blind Randomized Placebo-controlled Trial
Source: Schizophr Bull. 2026 May 9;52(3):sbag050. doi: 10.1093/schbul/sbag050 (PMC13156506; doi:10.1093/schbul/sbag050)
Supplement: sbag050_Supplementary_material [file sbag050_supplementary_material.docx]

**Table S1*. Inclusion and Exclusion Criteria***

| Inclusion Criteria | Exclusion Criteria |
| --- | --- |
| Age 18-64 years;  Met the criteria for schizophrenia and other psychotic disorders according to the Chinese-bilingual Structured Clinical Interview for Diagnostic and Statistical Manual of Mental Disorders, Fourth Edition Axis I (SCID-I), patient version, except for psychotic disorder due to a general medical condition and substance-induced psychotic disorder;  On at least one of the specified atypical antipsychotics – clozapine, olanzapine, quetiapine, risperidone or paliperidone, for a minimum of 6 months, with its dosage and concomitant psychiatric medications unchanged for the preceding month;  Asians with a BMI ≥ 23kg/m^2^;  Understand spoken instructions in Cantonese and read traditional Chinese;  Assessed to be clinically stable by their treating psychiatrists, with a Clinical Global Impression Scale- Severity (CGI-S) rating ≤ 4 (moderately ill);  Capable of giving informed consent. | Had a psychiatric diagnosis other than schizophrenia spectrum disorder as confirmed by SCID-I, learning disability, or active substance abuse or dependence  within the past six months (except nicotine);  Evidence of active liver, thyroid or renal dysfunction, cardiovascular disease, uncontrolled hypertension, diabetes mellitus or malignancy, as defined by:   1. Liver dysfunction: liver transaminases >3 times upper normal limit; 2. Renal dysfunction: estimated glomerular filtration rate (eGFR) <60 ml/min/1.73m^3^; 3. Cardiovascular disease: decompensated heart failure (New York Heart Association (NYHA) class III or IV), unstable angina pectoris and/ or myocardial infarction within the last 12 months; 4. Uncontrolled hypertension: systolic blood pressure >180mmHg or diastolic blood pressure >100mmHg; 5. Diabetes mellitus: glycated hemoglobin A1c (HbA1c) >6.4% (conventional unit);   History of using any medication for weight loss within the month preceding  study entry;  On oral corticosteroids;  Had recurrent urinary tract infection, defined as ≥2 urinary tract infections in past |

**Table S2. *Effects of physical activity levels and average daily caloric intake***

|  | Group | Time | Interaction (Time x Group) |
| --- | --- | --- | --- |
|  | F (p-value) | F (p-value) | F (p-value) |
| Physical activity levels, MET-minutes/week | 0.60 (.443) | **10.23** **(.002*)** | 46.59 (.896) |
| Average daily caloric intake, kcal | 2.25 (.141) | 0.05 (.826) | 0.83 (.367) |

*p<0.05

**Table S3. *Changes in End Points of physical activity levels and average daily caloric intake From Baseline to Week 16***

| Variables | SGLT2i group  (n = 26) | Placebo group  (n = 26) | SGLT2i group – placebo group  Estimated mean difference  [95% CI] | p-value |
| --- | --- | --- | --- | --- |
| Physical activity levels, MET-minutes/ week | 209.31  [-121.66, 540.28] | 349.16  [26.59, 671.74] | -139.85  [-604.20, 324.50] | .550 |
| Average daily caloric intake, kcal | 81.18  [-108.80, 271.16] | -18.61  [-204.11, 166.90] | 99.79  [-165.74, 365.31] | .457 |

**Table S4. *Effects and changes in end points of outcome measures after excluding outliers***

| Variables | Group Effect  F (p-value) | SGLT2i group – placebo group  Estimated mean difference  [95% CI] | p-value |
| --- | --- | --- | --- |
| Body weight, kg | No outliers | | |
| BMI, kg/m^2^ |  |  |  |
| Waist circumference, cm |  |  |  |
| Systolic blood pressure, mmHg |  |  |  |
| Diastolic blood pressure, mmHg |  |  |  |
| Fasting plasma glucose, mmol/L |  |  |  |
| HbA1c, % |  |  |  |
| LDL, mmol/L | 1.96 (.169) | 0.18 [-0.08, 0.43] | .169 |
| HDL, mmol/L | **4.82 (.033*)** | **0.10 [0.01, 0.18]** | **.033*** |
| TG, mmol/L | 1.67 (.203) | -0.21 [-0.55, 0.12] | .203 |

*p <0.05

**Table S5. *Changes in End Points from Baseline to Week 16 after including additional covariates***

| Variables | SGLT2i group  (n = 26) | Placebo group  (n = 26) | SGLT2i group – placebo group  Estimated mean difference  [95% CI] | p-value |
| --- | --- | --- | --- | --- |
| Body weight, kg | -2.32  [-3.13, -1.52] | -0.68  [-1.45, 0.10] | -1.65  [-2.76, -0.53] | **.005*** |
| BMI, kg/m^2^ | -0.80  [-1.09, -0.52] | -0.24  [-0.52, 0.03] | -0.56  [-0.96, -0.16] | **.007*** |
| Waist circumference, cm | -2.66  [-4.49, -0.84] | -2.34  [-4.09, -0.59] | -0.32  [-2.86, 2.22] | .800 |
| Systolic blood pressure, mmHg | -3.80  [-9.48, 1.88] | -5.08  [-10.59, 0.44] | 1.27  [-6.63, 9.17] | .749 |
| Diastolic blood pressure, mmHg | -0.93  [-4.46, 2.60] | -4.44  [-7.86, -0.01] | 3.51  [-1.41, 8.42] | .159 |
| Fasting plasma glucose, mmol/L | -0.41  [-0.60, -0.23] | -0.01  [-0.20, 0.17] | -0.40  [-0.66, -0.14] | .003* |
| HbA1c, % | -0.01  [-0.08, 0.06] | 0.08  [0.01, 0.14] | -0.09  [-0.18, 0.01] | .076 |
| LDL, mmol/L | -0.15  [-0.37, 0.07] | -0.22  [-0.43, -0.01] | 0.07  [-0.24, 0.38] | .642 |
| HDL, mmol/L | 0.03  [-0.04, 0.10] | -0.02  [-0.09, 0.04] | 0.05  [-0.05, 0.15] | .327 |
| TG, mmol/L | -0.06  [-0.36, 0.24] | 0.03  [-0.26, 0.32] | -0.09  [-0.51, 0.32] | .658 |

*p <0.05

**Table S6.** *Changes in End Points from Baseline to Week 16 in Prediabetes VS Normal*

|  | Prediabetes^a^ | | | | Normal | | | |
| --- | --- | --- | --- | --- | --- | --- | --- | --- |
| Variables | SGLT2i Group (n = 17)  [SE] | Placebo Group  (n = 22)  [SE] | SGLT2i group – placebo group  Estimated mean difference  [95% CI] | p-value | SGLT2i Group (n = 9)  [SE] | Placebo Group  (n = 4)  [SE] | SGLT2i group – placebo group  Estimated mean difference  [95% CI] | p-value |
| Body weight, kg | -2.32  [0.47] | -0.35  [0.42] | -1.96  [-3.23, -0.69] | **.003*** | -2.28  [0.50] | -2.45  [0.67] | +0.17  [-1.72, 2.06] | .844 |
| BMI, kg/m^2^ | -0.81  [0.17] | -0.13  [0.15] | -0.68  [-1.14, -0.22] | **.004*** | -0.76  [0.19] | -0.93  [0.25] | +0.17  [-0.51, 0.85] | .593 |
| Waist circumference, cm | -2.69  [1.09] | -1.51  [0.97] | -1.18  [-4.13, 1.77] | .425 | -2.64  [1.21] | -6.13  [1.60] | +3.49  [-1.25, 8.22] | .131 |
| Systolic blood pressure, mmHg | -4.06  [3.61] | -7.63  [3.23] | +3.57  [-6.20, 13.33] | .467 | -0.63  [3.41] | +2.85  [4.53] | -3.48  [15.54, 8.58] | .551 |
| Diastolic blood pressure, mmHg | -0.04  [2.19] | -4.15  [1.96] | +4.11  [-1.78, 10.00] | .168 | -2.71  [2.29] | -4.76  [3.04] | +2.05  [-6.17, 10.27] | .602 |
| Fasting plasma glucose, mmol/L | -0.46  [0.12] | -0.09  [0.11] | -0.38  [-0.70, -0.05] | **.025*** | -0.24  [0.11] | +0.20  [0.15] | -0.44  [-0.90, -0.02] | .041* |
| HbA1c, % | -0.06  [0.04] | +0.06  [0.04] | -0.12  [-0.24, -0.01] | **.032*** | +0.10  [0.05] | +0.11  [0.06] | -0.01  [-0.19, 0.16] | .852 |
| LDL, mmol/L | +0.01  [0.11] | -0.25  [0.10] | +0.26  [-0.05, 0.58] | .097 | -0.54  [0.20] | -0.06  [0.27] | -0.48  [-1.26, 0.30] | .192 |
| HDL, mmol/L | 0.07  [0.05] | -0.02  [0.04] | +0.09  [-0.04, 0.21] | .156 | -0.05  [0.06] | -0.04  [0.08] | -0.01  [-0.25, 0.23] | .909 |
| TG, mmol/L | -0.05  [0.17] | -0.01  [0.16] | -0.04  [-0.51, 0.43] | .856 | -0.04  [0.19] | 0.13  [0.25] | -0.17  [-0.89, 0.56] | .610 |

^a^ Prediabetes is defined as having either fasting plasma glucose of 5.6 – 6.9 mmol/L or HbA1c of 5.7-6.4% according to the American Diabetes Association (ADA).^47^*p <0.05

**Table S7.** *Changes in End Points from Baseline to Week 16 in Obese VS Overweight group*

|  | Obese | | | | Overweight | | | |
| --- | --- | --- | --- | --- | --- | --- | --- | --- |
| Variables | SGLT2i Group  (n = 20)  [SE] | Placebo Group  (n = 20)  [SE] | SGLT2i group – placebo group  Estimated mean difference  [95% CI] | p-value | SGLT2i Group  (n = 6)  [SE] | Placebo Group  (n = 6)  [SE] | SGLT2i group – placebo group  Estimated mean difference  [95% CI] | p-value |
| Body weight, kg | -2.35  [0.47] | -0.56  [0.45] | -1.79  [-3.11, -0.47] | **.009*** | -2.06  [0.66] | -1.18  [0.66] | -0.88  [-3.00, 1.24] | .376 |
| BMI, kg/m^2^ | -0.80  [0.17] | -0.21  [0.16] | -0.59  [-1.07, -0.11] | **.016*** | -0.73  [0.23] | -0.40  [0.23] | -0.33  [-1.09, 0.42] | .348 |
| Waist circumference, cm | -2.01  [1.01] | -2.31  [0.97] | +0.30  [-2.56, 3.16] | .832 | -3.27  [1.16] | -3.33  [1.16] | +0.07  [-3.54, 3.67] | .968 |
| Systolic blood pressure, mmHg | -3.75  [3.16] | -4.81  [3.06] | +1.06  [-7.74, 9.87] | .810 | -2.95  [6.15] | -7.22  [6.15] | +4.27  [-15.36, 23.91] | .640 |
| Diastolic blood pressure, mmHg | -2.24  [2.00] | -2.59  [1.94] | +0.35  [-5.24, 5.94] | .900 | +1.46  [2.97] | -7.63  [2.97] | +9.08  [-0.18, 18.35] | .054 |
| Fasting plasma glucose, mmol/L | -0.38  [0.10] | +0.03  [0.10] | -0.41  [-0.70, -0.12] | **.007*** | -0.54  [0.19] | -0.19  [0.19] | -0.35  [-0.96, 0.25] | .219 |
| HbA1c, % | -0.03  [0.04] | +0.09  [0.04] | -0.12  [-0.23, -0.01] | **.033*** | -0.05  [0.07] | +0.09  [0.07] | -0.14  [-0.38, 0.09] | .208 |
| LDL, mmol/L | -0.14  [0.13] | -0.24  [0.12] | +0.10  [-0.27, 0.47] | .586 | -0.17  [0.20] | -0.17  [0.20] | -0.00  [-0.65, 0.64] | .997 |
| HDL, mmol/L | +0.03  [0.04] | -0.01  [0.04] | +0.04  [-0.09, 0.16] | .541 | +0.08  [0.05] | -0.09  [0.05] | +0.17  [0.00, 0.34] | .046* |
| TG, mmol/L | -0.05  [0.16] | +0.15  [0.15] | -0.20  [-0.64, 0.24] | .373 | -0.17  [0.25] | -0.30  [0.25] | +0.13  [-0.69, 0.95] | .737 |

*p <0.05

**Table S8.** *Changes in End Points From Baseline to Week 16 in Olanzapine/Clozapine VS Other Antipsychotics group*

|  | Olanzapine/Clozapine | | | | Other Antipsychotics^a^ | | | |
| --- | --- | --- | --- | --- | --- | --- | --- | --- |
| Variables | SGLT2i Group  (n = 15)  [SE] | Placebo Group  (n = 19)  [SE] | SGLT2i group – placebo group  Estimated mean difference  [95% CI] | p-value | SGLT2i Group  (n = 11)  [SE] | Placebo Group  (n = 7)  [SE] | SGLT2i group – placebo group  Estimated mean difference  [95% CI] | p-value |
| Body weight, kg | -2.07  [0.47] | -0.77  [0.42] | -1.30  [-2.59, -0.01] | **.048*** | -2.51  [0.70] | -0.77  [0.80] | -1.74  [-3.97, 0.50] | .120 |
| BMI, kg/m^2^ | -0.72  [0.17] | -0.30  [0.15] | -0.42  [-0.89, 0.52] | .078 | -0.89  [0.24] | -0.19  [0.28] | -0.70  [-1.48, 0.07] | .073 |
| Waist circumference, cm | -2.91  [1.17] | -2.10  [1.05] | -0.81  [-4.02, 2.39] | .610 | -2.08  [1.35] | -3.05  [1.53] | +0.97  [-3.33, 5.27] | .642 |
| Systolic blood pressure, mmHg | -0.37  [3.13] | -1.62  [2.85] | +1.25  [-7.22, 9.72] | .769 | -9.29  [4.95] | -15.03  [5.61] | +5.74  [-9.84, 21.32] | .451 |
| Diastolic blood pressure, mmHg | +0.69  [2.00] | -1.68  [1.82] | +2.37  [-3.08, 7.81] | .387 | -3.72  [2.90] | -10.75  [3.28] | +7.03  [-2.00, 16.05] | .121 |
| Fasting plasma glucose, mmol/L | -0.50  [0.10] | -0.07  [0.09] | -0.44  [-0.72, -0.15] | **.004*** | -0.13  [0.18] | -0.09  [0.21] | -0.04  [-0.72, 0.64] | .898 |
| HbA1c, % | -0.04  [0.04] | +0.10  [0.04] | -0.14  [-0.25, -0.02] | **.020*** | -0.03  [0.07] | +0.06  [0.08] | -0.09  [-0.30, 0.13] | .397 |
| LDL, mmol/L | -0.05  [0.13] | -0.16  [0.12] | +0.12  [-0.24, 0.47] | .512 | -0.35  [0.19] | -0.35  [0.21] | +0.00  [-0.62, 0.63] | .991 |
| HDL, mmol/L | +0.01  [0.06] | -0.10  [0.06] | +0.10  [-0.04, 0.23] | .150 | +0.06  [0.06] | +0.03  [0.07] | +0.03  [-0.17, 0.23] | .764 |
| TG, mmol/L | -0.18  [0.15] | +0.09  [0.14] | -0.27  [-0.69, 0.16] | .209 | -0.01  [0.30] | +0.06  [0.35] | -0.07  [-1.10, 0.96] | .886 |

^a^ Other Antipsychotics include participants who are not taking olanzapine/ clozapine and are on risperidone/ paliperidone/ quetiapine.
*p <0.05
